# Annotations

**Published:** 1909-08-21

**Authors:** 


### ANNOTATIONS.

### Cinematographs and the Public.

The unfortunate panic which occurred at a cinematographic display at Southsea this week will help to draw attention to the urgent necessity for safeguarding the public interests in places where such entertainments are given. An attempt has been made by the present Government to deal with the matter in the Cinematograph Act which was passed on August 9, and which is shortly to be enforced. The Southsea disaster comes aptly—though one could easily have missed so unlucky a reminder—to urge the arguments in favour of this short, concise and wholesome little new Act which makes what appears to be adequate provision for the public safety. By its terms cinematographic displays, or indeed any displays which involve the use of inflammable films or dangerous material, can only be given in properly licensed rooms. Such rooms must be inspected by the local authorities, who have powers under this Act to carry out its provisions. In cases where entertainments are not habitually given, but where the display is only taking place for "once in a while," as the saying is, due notice of such entertainment must be given to the local authority, whose sanction must be obtained before the entertainment can be begun. These provisions seem to us to be adequate and to provide the necessary safeguards, so far as it is possible to provide for such. At present many of these "shows" are veritable death traps in case of fire or a panic, and the wonder is that there have been so few disasters so far.

### Medical Candour.

Medical men are often asked by their lay friends for an expression of semi-professional opinion on matters which are popularly supposed to be semi-scientific. However flattering such requests may be, they have a sad side in so far as an open and honest expression of the practitioner's candid thoughts upon the subject not infrequently alienates the friendship of his lay acquaintance. In our editorial capacity we have on many occasions received similar requests, not always from lay readers, and we have doubtless been accused of partisanship, prejudice, and all the mental obstinacy of a Turkish seraskier, for having editorially been incautious enough to believe in the "good natured tolerance of all men." The awful example of Gil Blas, whose candour in reply to the Bishop's cordial invitation to express an opinion on the episcopal pulpit methods, led to his summary ejection from the palace, should serve as a warning even if we had not examples of our own, as every practitioner has, to refer to. But there are times and occasions when plain speaking becomes a duty which medical men, collectively or individually, cannot and may not shirk. There are many popular fallacies of the day—fallacies which are the more mischievous in proportion as they are held more honestly—which demand exposure and confutation. These fallacies it is the duty of the medical man, once they are referred to him for an expression of opinion, emphatically to deal with, even at the risk of being misunderstood, misquoted, and misinterpreted by the laity. It is therefore with considerable pleasure that we have noticed in a lay contemporary a medical man's protest against the fallacy of what Sir James Crichton Browne is pleased to call "Parcimony in Nutrition." The simple life may be carried too far, and we are not all Cornaros, nor likely to be if Chittenden's subjects expressed themselves so strongly on the subject of spare diet as they are alleged to have done. We are told, in fact, that many of the subjects who have been vaunted as "perfect examples of the possibility of living on a minus daily diet" told impartial investigators that they consoled themselves on the sly by lunching when they were supposed to be starving, and consuming bacon and beans when their official ration was supposed to be a diet calculated on a more rigorous basis than Banting's or Valsalva's. We need not agree in everything with our professional friend who so bravely takes up the case for the pleasures of the table, but all diet reformers must admit that there is a great deal of common sense in his remarks. At any rate it is high time for someone professionally to protest against the simple life carried to an absurdity and moderation in diet brought to the verge of semi-starvation.

### Meat Preservation.

The recently issued report to the Local Government Board on the subject of the application of formaldehyde to meat, presented by Drs. Buchanan and Schryver, contains many points of interest. The use of formaldehyde as a preservative has of late been increasing in the trade, particularly in the case of chilled meat brought from South American ports to this country. Meat safes fitted with arrangements by which meat deposited in them can be "fumigated" with formaldehyde are becoming popular with provision merchants, and the various solutions containing the preservative are advertised as being "non-acid and non-poisonous." The examiners appointed by the Local Government Board appear to have gone very fully into the matter, and their conclusions are that from a sanitary point of view no objection can be taken to the formalin treatment (fumigation) of chilled meat in so far as "it forms a satisfactory and convenient method of disinfecting and sterilising the hold in which the meat is placed during the voyage." At the same time the examiners insist that there are substantial and well-founded reasons for objecting to the presence of formaldehyde in food stuffs. It is a very powerful disinfectant; even comparatively small quantities materially affecting the digestibility of meat by combining with the proteid and forming a less digestible compound. For these reasons the Departmental Committee in 1901 recommended its total prohibition as a food preservative. The examiners conclude their report by advising meat importers and traders to limit the use of formalin to the adequate disinfection of the holds before the meat is introduced.

